# MEO based secured, robust, high capacity and perceptual quality image watermarking in DWT-SVD domain

**DOI:** 10.1186/s40064-015-0904-z

**Published:** 2015-03-14

**Authors:** Baisa L Gunjal, Suresh N Mali

**Affiliations:** Department of Computer Engineering, Padmashree Dr. D.Y. Patil Institute of Engineering and Technology, Pune, 411018 India; Sinhgad Institute of Technology and Science, Narhe, Pune, 411041 India

**Keywords:** MEO, FLT, Histogram, Multiobjective, Robustness, Optimization and wavelet

## Abstract

The aim of this paper is to present multiobjective evolutionary optimizer (MEO) based highly secured and strongly robust image watermarking technique using discrete wavelet transform (DWT) and singular value decomposition (SVD). Many researchers have failed to achieve optimization of perceptual quality and robustness with high capacity watermark embedding. Here, we achieved optimized peak signal to noise ratio (PSNR) and normalized correlation (NC) using MEO. Strong security is implemented through eight different security levels including watermark scrambling by Fibonacci-Lucas transformation (FLT). Haar wavelet is selected for DWT decomposition to compare practical performance of wavelets from different wavelet families. The technique is non-blind and tested with cover images of size 512x512 and grey scale watermark of size 256x256. The achieved perceptual quality in terms of PSNR is 79.8611dBs for Lena, 87.8446 dBs for peppers and 93.2853 dBs for lake images by varying scale factor K1 from 1 to 5. All candidate images used for testing namely Lena, peppers and lake images show exact recovery of watermark giving NC equals to 1. The robustness is tested against variety of attacks on watermarked image. The experimental demonstration proved that proposed method gives NC more than 0.96 for majority of attacks under consideration. The performance evaluation of this technique is found superior to all existing hybrid image watermarking techniques under consideration.

## Introduction

We are living in era of information technology with internet and mobile phones where billions of bits of multimedia data including images, audios, videos, digital libraries, online transactions are created, copied and transmitted in every fraction of second. Majority of transactions like railway, airplane reservations, shopping, banking, submitting tax returns are done online. The deployment of information and communication technology infrastructure is bringing revolution to health industry. The administration of Obama is offering $ 44,000 to $ 64,000 for electronic medical record (EMR) system based medical practices (Kamran & Farooq 2012). The unauthorized replication problem is critical issue. Digital image watermarking provides copyright protection by hiding appropriate ownership information in digital images. Thus, it is essentially required as value-added technique for providing authentication features (Kamran & Farooq 2012). Robustness, imperceptibility, embedding capacity and security are four essential attributes those commonly determine quality of watermarking scheme (Urvoy et al. 2014). The main challenge in digital image watermarking is to achieve these parameters simultaneously as they conflict each other while achieving multiple level security. The spatial domain technique dual intermediate significant bit (DISB) is presented in (Mohammed et al. 2014) but it is vulnerable against simple attack. Most of the existing watermarking algorithms are developed in transform domain (Lai & Tsai 2010). In transform domain, watermark is inserted into transformed coefficients of image giving more information hiding capacity and more robustness against watermarking attacks because information can be spread out to entire image. The fragile watermarking techniques are given in (Piper & Safavi-Naini 2013; Serra-Ruiz & Megias 2010). A blind watermarking technique for 3D images is presented in (Lin & Wu 2011). Spread spectrum (SS) watermarking based watermarking schemes are given in (Ehsan Nezhadarya et al. 2011; Kuribayashi 2014). In SS based watermarking, adding pseudo random noise like watermark into host signal is found robust in many attacks. In quantization based watermarking, set of features extracted from host signal are quantized so that each watermark bit is represented by quantized feature value. Reversible watermarking is special data hiding technique where original digital content can be completely restored after data extraction (Li et al. 2011; Coatrieux et al. 2009). Majority of watermarking techniques are invisible. The visible watermarking with exact recovery of cover image is presented in (Liu & Tsai 2010), however visible methods are used in limited application areas. Lossless data embedding methods are presented in (Shi et al. 2004; Celik et al. 2006). Secured image data transmission is required in applications such as teleradiology, telepathy, telecare, telesurgery, teleneurology demand safety and confidentiality of medical. The watermarking based security for telemedicine is provided in methods (Kamran & Farooq 2012; Bouslimi et al. 2012; Coatrieux et al. 2009). Many researchers have used specific transforms for implementing watermarking schemes. Most commonly used transforms are DFT, discrete Cosine transforms (DCT), discrete Laguerre transform (DLT), discrete Hadamard transform (DHT) and DWT. The DWT based watermarking methods are presented in (Wang et al. 2002; Abu-Errub & Al-Haj 2008; C-q et al. 2007; Aslantas et al. 2008; Senthil & Bhaskaran 2008). Fourier transform based methods are given in (Tsui et al. 2008; Tsui et al. 2006a,b). DWT-SVD based watermarking algorithms are proposed in (Lai & Tsai 2010; Ganic & Eskicioglu 2004; C-q et al. 2007; Singh et al. 2012; Wang & Kim 2009). The Redundant DWT-SVD based method is presented in (Lagzian et al. 2011). Contourlet transform and DCT are effectively combined using local complexity variations as given in (Azizi et al. 2013). Some researchers tried to optimize perceptual transparency and robustness under high payload scenario with the help of optimization techniques. Wavelet-based genetic algorithm (GA) method (Ramanjaneyulu & Rajarajeswari 2012), redundant DWT-SVD (RDWT-SVD) based optimizer (Lagzian et al. 2011), DWT-SVD based particle swarm optimizer (Aslantas et al. 2008) are example GA based techniques. The DCT has special energy compaction property. Most of visually significant information of the image is concentrated in just a few coefficients of the DCT. The DCT based methods are given in (Wei & Ngan 2009; Ahumada & Peterson 1992). Some of the researchers have done experimentation by combining DCT with other transforms. The combine DWT-DCT approach is used in (Nikolaidis & Pitas 2003), DWT-DCT-SVD approach is used in (Sivavenkateswara et al. 2012). Image scrambling is used for secured watermark embedding. Different researchers have used various scrambling methods like Fibonacci transformation (Zou et al. 2004a), modified Fibonacci transform (Zou et al. 2004a), generalized Fibonacci transform (Zou et al. 2004b), Arnold transform (Umamageswari & Suresh 2013), grey code transformation (Zou et al. 2005), affine modular transform (Ehsan Nezhadarya et al. 2011). Other watermark scrambling based methods are presented in (Zou et al. 2005).

The most of researchers have been failed to develop effective watermarking techniques to fulfill four quality parameters simultaneously namely robustness, imperceptibility, high capacity watermark embedding and security. The novelty of proposed MEO based technique is to optimize imperceptibility and robustness in DWT-SVD domain under high payload scenario with strong security provision.

## Theory and mathematical background

### Wavelet selection in DWT implementation

Single level decomposition of signal using discrete wavelet transform is given by (Sidney Burrus et al. 2008),

1 $$ {Y}_{high}(n)={\displaystyle \sum_{k=-\infty}^{\infty }}x(k)g\left(2n-k\right) $$

2 $$ {Y}_{low}(n)=\kern0.5em {\displaystyle \sum_{k=-\infty}^{\infty }}x(k)h\left(2n-k\right) $$

Where, *x*(*k*) is signal, *g*(*k*) and *h*(*k*) are high pass filter and low pass filter respectively. *Y*_high_(*n*) and *Y*_low_(*n*) are outputs of low pass and high pass filters respectively after subsampling by 2. While reconstructing, signals at every level are upsampled by two, passed through synthesis filters, *h*^'^(*n*) and *g*^'^(*n*) respectively and then added.

3 $$ x(k)={\displaystyle \sum_{n=-\infty}^{\infty }}\left(\ {Y}_{high}(n){g}^{\hbox{'}}\left(2n-k\right)\right) + {\displaystyle \sum_{-\infty}^{\infty }}\left(\ {Y}_{low}(n){h}^{\hbox{'}}\left(2n-k\right)\right) $$

Image itself is considered as two dimensional signals. In the case of DWT, the mother wavelet is expressed as (Singh et al. 2012)

4 $$ {\psi}_{j,k}(t)={a}_0^{-j/2}\psi\ \left({a}_0^{-j}t-k\ {b}_0\right) $$

Where, *ψ*, is mother wavelet, *a*_0_ is scale parameter, *b*_0_ is translation parameter. For dyadic wavelets a_0_ = 2 and b_0_ = 1, Thus, we have,

5 $$ {\psi}_{j,k}(t)={2}^{-j/2}\psi\ \left({2}^{-j}t-k\ \right)\cdots \mathrm{j},\ \mathrm{k}\ \epsilon\ Z $$

**Figure 1:**
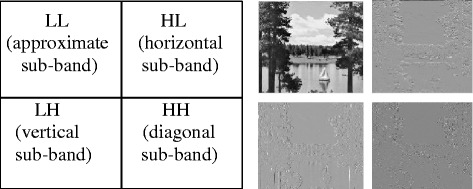
**One level DWT decomposition of la0ke image.**

When series of low pass and high pass filters are applied to an image, DWT decomposes it into sub-bands of different resolutions. DWT provides multiresolution representation of image and gives perfect reconstruction of image. The one level decomposition of image can be achieved by scanning it horizontally and then by scanning vertically. The one level DWT gives four non overlapping sub-bands namely LL (approximate sub-band), HL (horizontal sub-band), LH (vertical sub-band) and HH (diagonal Sub-band) as shown in Figure 1. DWT can be applied at different levels (Wang et al. 2002).

The use of multiobjective optimization using genetic algorithm is major part of this paper. Genetic algorithms are relatively slow as they are iterative in nature. Thus time complexity is critical issue of GA based algorithms. In GA process our objective is to select wavelet that will give better performance with less amount of time period.

**Figure 2:**
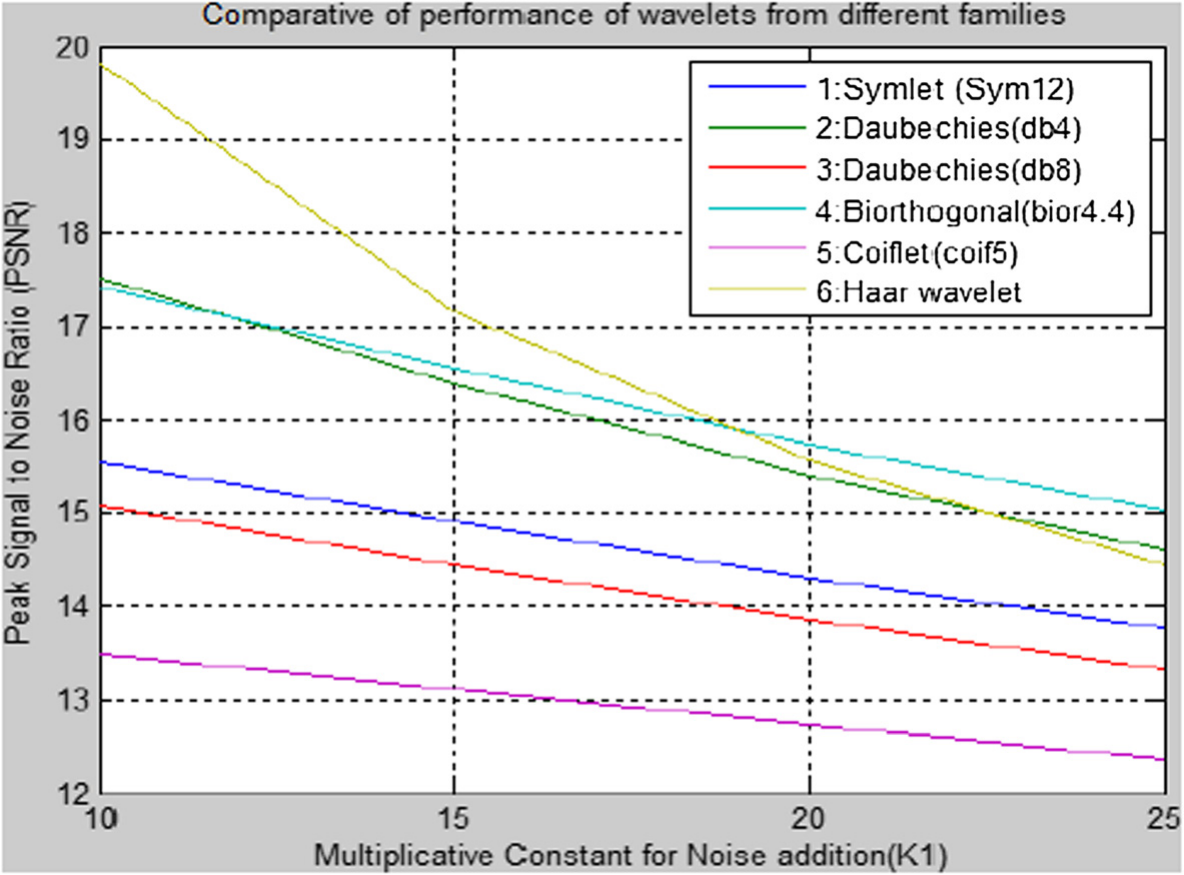
**Comparative of performance of wavelets from different families.**

The numbers of tests are carried out for selection of wavelet by considering comparative performance of wavelets from different wavelet families. The sample test results for Lena image are given in Figure 2 and Table 1. Here, Lena image of 512x512 sizes was decomposed with one level DWT using different wavelets separating, LL, HL, LH and HH sub-bands and then noise is added in HL sub-band using equation (6),

6 $$ Ne{w}_{HL}=K1*HL $$

Where, K1 multiplicative constant called scale factor is varied from 10 to 25. The noisy image is composed using LL, *New*_HL_, LH and HH sub-bands.

**Table 1:** **Comparative computation time (unit seconds) with different wavelets**

**Wavelet**	**K1 = 10**	**K1 = 15**	**K1 = 20**	**K1 = 25**
Symlet (Sym12)	0.9984	0.9672	0.9828	1.0296
Daubechies (db4)	0.8580	0.7488	0.8424	0.7332
Daubechies (db8)	0.8736	0.9204	0.9672	0.8892
Biorthogo-nal(bior4.4)	0.8424	0.9204	0.8112	0.9204
Coiflet (coif5)	1.1544	1.1076	1.0296	1.1388
Haar	0.7800	0.8580	0.7800	0.9204

The PSNR between original cover image and noisy image is calculated. The execution time i.e. computation time is noted with different wavelets namely, Symlet (Sym12), Daubechies (db4 and db8), Biorthogonal (bior4.4), Coiflet (coif5) and Haar wavelet. It is found that Haar wavelet gives better PSNR with compared to other wavelets depending on value of K1.

The comparative computation time of this algorithm is minimum with Haar i.e.0.7800 for K1 = 10 and K1 = 20. Hence Haar wavelet has been selected for DWT decomposition in proposed methodology based on this performance. The Haar is simple, symmetric and orthogonal wavelet.

### Selection of wavelet coefficients

The addition of watermark is equivalent to addition of noise to the cover image. Hence, the selection of coefficient for watermark embedding is very critical task. The images have maximum energy associated with low frequency sub-bands. Hence, watermark embedding in frequency sub-band (LL) should be avoided as it directly affects perceptual quality of image. The human naked eyes cannot detect modifications in high frequency coefficients. However high frequency sub-band(HH) contains edges and texture information of the image. In fact, high frequency coefficients are removed at the time of image compression which is normally applied before image transmission. Thus, rest of the choices is middle frequency sub-bands (HL and LH). But human visual system (HVS) is less sensitive in horizontal than vertical (Singh et al. 2012). Hence, HL has been selected for watermark embedding in proposed work.

### SVD

SVD is used for variety of image processing applications such as image watermarking, image steganography, image compression, noise reduction. SVD is important linear algebraic technique that used to solve many mathematical problems. SVD of an image A with size MxN is represented as A = U∑V^T^ , where U and V are orthogonal matrices such that, UU^T^ = I and VV^T^ = I , ∑ is summation of diagonal entries λ_1_, λ_2_…..gives the singular vectors of A. These diagonal entries are called as singular values of A and the decomposition is called as ‘singular value decomposition’. Thus we have (Lai & Tsai 2010),

7 $$ \mathrm{A}={\uplambda}_1{\mathrm{U}}_1\ {\mathrm{V}}_1^{\mathrm{T}}+{\uplambda}_2{\mathrm{U}}_2\ {\mathrm{V}}_2^{\mathrm{T}}+\dots ..+{\uplambda}_{\mathrm{r}}{\mathrm{U}}_{\mathrm{r}}\ {\mathrm{V}}_{\mathrm{r}}^{\mathrm{T}} $$

Where, r is rank A. The columns of U and V are called left and right singular vectors of B. The singular values have following properties,

i. Singular values correspond to brightness. The left singular and right singular vectors reflect geometric characteristics of image.
ii. The slight variations in singular values do not affect much visual perception.
iii. B and *B*_r_ (B rotated by certain degree) have same nonzero singular values.
iv. The row flipped *B*_rf_ and column flipped *B*_cf_ forms of B have same nonzero singular values.
v. If *B*_e_ is expanded by adding rows and columns of black pixels, the resulting *B*_e_ has same nonzero singular values of B.

### FLT

The scrambling methods transform meaningful image information into disorder and unsystematic pattern for hiding the real meaning. Various scrambling methods including Fass curve, gray code, magic square, Arnold transform, modified Arnold transform, Fibonacci-Q transform and generalized Fibonacci transforms are used by researchers in stenographic and watermarking techniques. But, these methods do not provide enough security. Here, Fibonacci-Lucas transform is proposed to scramble watermark before embedding into cover image. The French mathematician Lucas proposed Lucas-series, which is extended version of Fibonacci series (Zou et al. 2004a; Zou et al. 2004b). Fibonacci-Q transform is given by,

8 $$ \left(\begin{array}{c}\hfill {X}_2\hfill \\ {}\hfill {Y}_2\hfill \end{array}\right)=\left[\begin{array}{cc}\hfill 1\hfill & \hfill 1\hfill \\ {}\hfill 1\hfill & \hfill 0\hfill \end{array}\right]\left(\begin{array}{c}\hfill {X}_1\hfill \\ {}\hfill {Y}_1\hfill \end{array}\right)\left( mod\ M\right) $$

Where, (_X1_, *Y*_1_) = {0,1,.....} are pixel coordinates of original image (*X*_2_, *Y*_1_) are transformed coefficients after applying Fibonacci-Q transform. M is size of original image. The generalized Fibonacci- transform is given by,

9 $$ {T}_m=\left\{\begin{array}{c}\hfill \begin{array}{cc}\hfill 0\hfill & \hfill \kern4.5em  if\ m=0\hfill \end{array}\hfill \\ {}\hfill \begin{array}{cc}\hfill 1\hfill & \hfill \kern4.5em  if\ m=2\ \hfill \end{array}\hfill \\ {}\hfill \begin{array}{cc}\hfill {F}_{m-1} + {F}_{m-2\kern0.75em }\hfill & \hfill Otherwise\hfill \end{array}\hfill \end{array}\right. $$

Using Fibonacci series various sequences of numbers can be generated as,

- Fibonacci-12: 1, 2, 3, 5, 8, 13, 21,…..
- Fibonacci-23: 2, 3, 5, 8, 13, 21, 34 , ….
- The Lucas series is non-periodic series is given by,

10 $$ {S}_m=\left\{\begin{array}{c}\hfill \begin{array}{cc}\hfill 2\hfill & \hfill \kern4.5em  if\ m=1\hfill \end{array}\hfill \\ {}\hfill \begin{array}{cc}\hfill 1\hfill & \hfill \kern4.5em  if\ m=2\ \hfill \end{array}\hfill \\ {}\hfill \begin{array}{cc}\hfill {L}_{m-1} + {L}_{m-2\kern0.75em }\hfill & \hfill Otherwise\hfill \end{array}\hfill \end{array}\right. $$

Combining Lucas series with Fibonacci transform, Fibonacci-Lucas transform is given by,

11 $$ \left(\begin{array}{c}\hfill {X}_2\hfill \\ {}\hfill {Y}_2\hfill \end{array}\right)=\left[\begin{array}{cc}\hfill {T}_i\hfill & \hfill {T}_{i+1}\hfill \\ {}\hfill {S}_i\hfill & \hfill {S}_{i+1}\hfill \end{array}\right]\left(\begin{array}{c}\hfill {X}_1\hfill \\ {}\hfill {Y}_1\hfill \end{array}\right)\left( mod\ M\right) $$

Where, (*X*_1_, *Y*_1_) = {0,1,.....M-1} are pixel coordinates of original image, *T*_i_ is the *i*^th^ term of Fibonacci series *S*_i_ is the *i*^th^ term of Lucas series, (i = 1, 2, 4, 5……….) (*X*_2_, *Y*_2_) are transformed coefficients after applying Fibonacci-Lucas transform. M is size of original image.

As, two transforms are combined, resulting Fibonacci-Lucas transform provides more security in watermark embedding phase.

### GA based optimization

Genetic algorithms are widely used to solve various problems in scientific and engineering applications (Ramanjaneyulu & Rajarajeswari 2012). The single objective based image watermarking process is given below through step-1 to step-4.

- **Step-1:** Initialize population size P, crossover rate Pc, mutation rate Pm, maximum generations N, scale factor K1.
- **Step-2:** Generate first generation of GA process using parameters in watermark embedding process. The different watermarked image is generated for each individual.
- **Step-3:** While generation ≤ N.
  i. Find perceptual quality of watermarked image computing its PSNR
  ii. Apply attack on watermarked image
  iii. Call watermark extraction process
  iv. Find robustness by computing normalized correlation between original watermark and extracted watermark.
  v. Do evaluation based on fitness function where,
  vi. Fitness Function = PSNR + K1*NC
  vii. Select individuals with the best fitness values.
  viii. Generate new population by performing crossover and mutation on the selected individuals.
  ix. End while
- **Step-4:** Display PSNR, NC.

GA process starts with randomly selected population called first generation. The individual in population is called chromosome and all possible chromosomes constitute the population. The objective function also called fitness function evaluates the quality of each chromosome and it measures degree of goodness of candidate solution. The chromosomes where fitness value is high are selected for future generation. The initial population, selection, crossover and mutation are major stages of GA process. GA uses reproduction, crossover, and mutation repeatedly until either a predefined criterion is satisfied or numbers of iterations are completed.

### MEO

Here, MEO in Matlab is used to optimize multiple objectives. Single objective optimization algorithms find single optimum solution for given fitness function. The goal of single objective optimization is to find global optima. While, minimizing one of the objective may not achieve desired effect on other. The aim of using MEO is to find optimum values of multiple objectives. In digital image watermarking, our goal is to achieve two optimized performance parameters perceptual transparency and robustness simultaneously against different attacks. We tried to achieve optimization of PSNR and NC for given scale factor (K1) with help of MEO.

### Objective function 1

This is to evaluate the perceptual quality of image. Perceptual transparency is perceived quality of image which should not be destroyed by presence of watermark. The quality of watermarked image is measured by PSNR. The bigger PSNR implies better perceptual quality of watermarked image. PSNR between two grey scale images *Image*1(*i*, *j*) and *Image*2(*i*, *j*) is given by (Kamran & Farooq 2012; Azizi et al. 2013; Ramanjaneyulu & Rajarajeswari 2012),

12 $$ PSNR(db)=10{ \log}_{10}\ \frac{Ma{x_I}^2}{\frac{1}{M*N}{\displaystyle {\sum}_{i=1}^M}{\displaystyle {\sum}_{j=1}^N}{\left[ Image1\left(i,j\right)- Image2\left(i,j\right)\right]}^2} $$

- Where, Max_I_ is 255 for grey scale image,
- Image1 (i, j) is pixel of original image,
- Image2 (i, j) is pixel values of watermarked image,
- M and N are the number of rows and columns both images.

### Objective function 2

This is to evaluate the robustness of watermarked image. Robustness is measure of susceptibility of watermark against attempts to remove or destroy it by image attacks such as noise addition, noise filtering, scaling, translation, resizing, cropping, blurring, compression, rotation, collision attacks. NC measures the similarity and difference between original watermark and extracted watermark. Ideally it should be 1 but value 0.75 is acceptable.

It is given by (Ganic & Eskicioglu 2004; Lagzian et al. 2011; Azizi et al. 2013; Sivavenkateswara et al. 2012),

13 $$ NC = \frac{{\displaystyle {\sum}_{i=1}^N} Watermar{k}_i\kern0.75em  Watermar{k}_i\ \hbox{'}}{\sqrt{{\displaystyle {\sum}_{i=1}^N} Watermar{k}_i}\kern0.5em \sqrt{{\displaystyle {\sum}_{i=1}^N} Watermar{k}_i}\ \hbox{'}} $$

Where, *Watermark*_i_ is original watermark, *Watermark*_i_ ' is extracted watermark, N is number of pixels in watermark.

## Proposed MEO based methodology in DWT-SVD domain

The proposed technique is implemented using eight different stages including cover object processing phase and watermark processing phase.

### Implementation of eight stage security

The eight stages used for security implementation are given in Table 2.

**Table 2:** **Applying multistage security**

**Stages**	**Detail description of given stage**
Stage-1:	Cover_Object is taken into Wavelet domain.
Stage-2:	As per given ‘State’ the Pn_Sequence of Watermark using ‘Key1’ is generated.
Stage-3:	Calculate AVG = average of Pn_Sequence
Stage-4:	Apply thresholding with ‘Key1’ to generate ‘K’ required for scrambling.
Stage-5:	Use Fibonacci-Lucas Transform for scrambling Watermark with K.
Stage-6:	Apply Singular Value Decomposition,
Stage-7:	Apply ‘Embedding Formula’ with given scale factor K1. This K1 will be used for optimization in Step-8.
Stage-8:	Apply MEO to optimize PSNR and NC using K1

### MEO based watermark embedding algorithm

Initially, pseudo random number sequence of watermark using Key1 at given state is generated. The average of pseudo random number sequence is computed. The key K is determined based on predefined threshold value T. This K is used for watermark scrambling using Fibonacci-Lucas transform. Practically, sample periodicity of Fibonacci-Lucas transform for M × N image with M = N is found as M. Here, for grey scale watermark images of size 256 × 256, the scrambling key 100 and descrambling key 156 are used as sample. K1 is scale factor which is used in MEO based watermark embedding algorithm. Here K, Key1 and K1 are integer values. The grey scale watermark, say W is scrambled to give scrambled watermark SW which is embedded in cover object by applying multiplicative rule. The MEO based watermark embedding algorithm is as follows,

**Input:** Cover_Object, Watermark W.

**Output:** Watermarked_Object.

- **Step-1:** Read grey scale Cover_ Object of size MxN.
- **Step-2:** Decompose Cover_ Object using Haar wavelet, [LL,HL,LH,HH] = dwt2(Cover_Object,'Haar');
- **Step-3:** Apply SVD to HL sub-band of Cover_Object found in step 2: [U,S,V] = SVD(HL)
- **Step-4:** Read grey scale watermark W of size 256x256.
- **Step-5:** As per state of watermark W, generate Key1. Generate Pn_Sequence with ‘key1’. Calculate AVG = average of Pn_Sequence.
- **Step-6:** Calculate K in step 7 using predefined threshold T, predefined counter Count , Fibonacci-Lucas periodicity P and Key1 generated in step 5.
- **Step-7:** Key1 + Count ≤ P, If AVG > T then K = P + Count else K = P-Count.
- **Step-8:** Generate scrambled watermark SW by applying Fibonacci-Lucas transform to Watermark with scrambling key K as per equation 11.
- **Step-9:** Perform embedding of watermark SW with Cover_Object by considering S found in step 3, SW found in step 8 and K1, S1 = S + K1*SW [U1,SS,V1] = SVD(S1)
- **Step10:** Apply inverse SVD to get New_HL component as: CWI = U*SS*V', New_HL = CWI
- **Step-11:** Now apply one level inverse DWT with New_HL component to form Watermarked_Object as, Watermarked_Object = idwt2(LL,New_HL,LH,HH,'Haar',[M,N]);
- **Step-12:** Display Cover_Object, Watermarked_Object, PSNR and K1.

### MEO based watermark extraction algorithm

The overall watermark extraction process is implemented using step-1 through step-10 as shown below.

**Input:** Watermarked_Object ,Cover_Object,

**Output:** Extracted_Watermark, NC.

- **Step-1:** Read Watermarked_Object
- **Step-2:** Apply One level DWT to Watermarked_Object to have Recovered_HL1 component as, [LL,Recovered_HL,LH,HH] = dwt2(Watermarked_Object, 'Haar');
- **Step-3:** Apply SVD to Recovered_HL as, [UU,S2,VV] = SVD( Recovered_HL).
- **Step-4:** Read grey scale Cover_Object, size MxN.
- **Step-5:** Apply one level DWT to Cover_Object using Haar wavelet to get LL,HL,LH,HH sub-bands, [LL,HL,LH,HH] = dwt2(Cover_Object,'Haar');
- **Step-6:** Apply SVD to HL sub-band of cover image found in step 2: [U,S,V] = SVD(HL)
- **Step-7:** Find SN using component S2 in step 3 and components: U1 and V1 in step 9 of watermark embedding algorithm,
- SNEW = U1*S2*V1'
- **Step-8:** Find Scrambled _Watermark using SN in step 7, S in step 6 and scale factor K1 used in watermark embedding algorithm as, Scrambled_Watermark = (SNEW -S)/K1.
- **Step-9:** Apply Fibonacci-Lucas transform to Scrambled_Watermark to find final Extracted_Watermark with key K.
- **Step-10:** Display Extracted_Watermark and NC.

### Implementation of with MEO based algorithm

The MEO based watermark embedding algorithm and MEO based watermark extraction algorithm are used in Trial(K1) function, where K1 is scale factor passed to the function using multiobjective evolutionary tool in Matlab. The PSNR, NC and optimized K1 are displayed as output of this function. The algorithmic steps of ‘Trial’ function are given below.

**Input:** K1 passed through MEO based GA process.

**Output:** PSNR, NC

- **Step-1: Specify range of scale factor K1.**
- **Step-2:** Specify GA parameters population size, reproduction rate, crossover rate and mutation rate.
- **Step-3:** Specify termination criteria by number of generations.
- **Step-4:** Write MEO based watermark embedding algorithm.
- **Step-5:** Apply attack on Watermarked_Object.
- **Step-6:** Write MEO based watermark extraction algorithm
- **Step-7:** Display parameters in step 8 at end of function as output.
- **Step-8:** Display y(1) = PSNR ,y(2) = NC.

## Experiments and results

The proposed technique is implemented using Matlab version 8.0.0.7837 (R2012b) with multiobjective evolutionary optimizer tool. The experimentation is carried out on Intel(R) Core(TM) i3 processor of 2.10 GHz and 2GB RAM with 64 bit windows operating system.

### Performance evaluation

**Figure 3:**
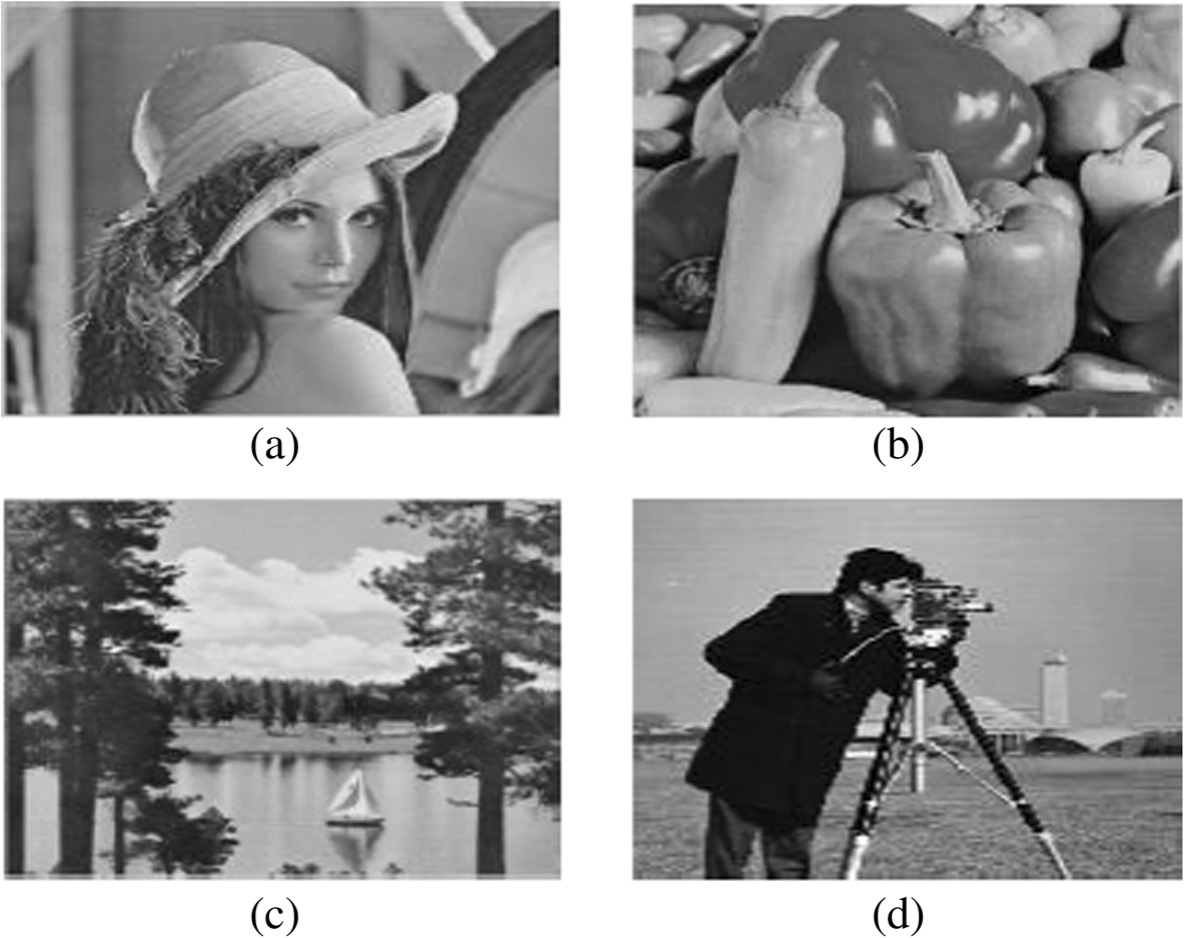
**Cover images of size 512 × 512 a) Lena (b) pepper c) cover image lake d) watermark of size 256 × 256.**

The performance evaluation is done to evaluate performance quality metrics given in equations 12 and 13. The cover images Lena, peppers and lake of sizes 512 × 512 and grey scale watermark cameraman of size 256 × 256 from standard online databases (CVG-UGR Image Database; OsiriX) as shown in Figure 3 are used for experimentation. The experimentation is carried out to evaluate imperceptibility and robustness simultaneously.

**Figure 4:**
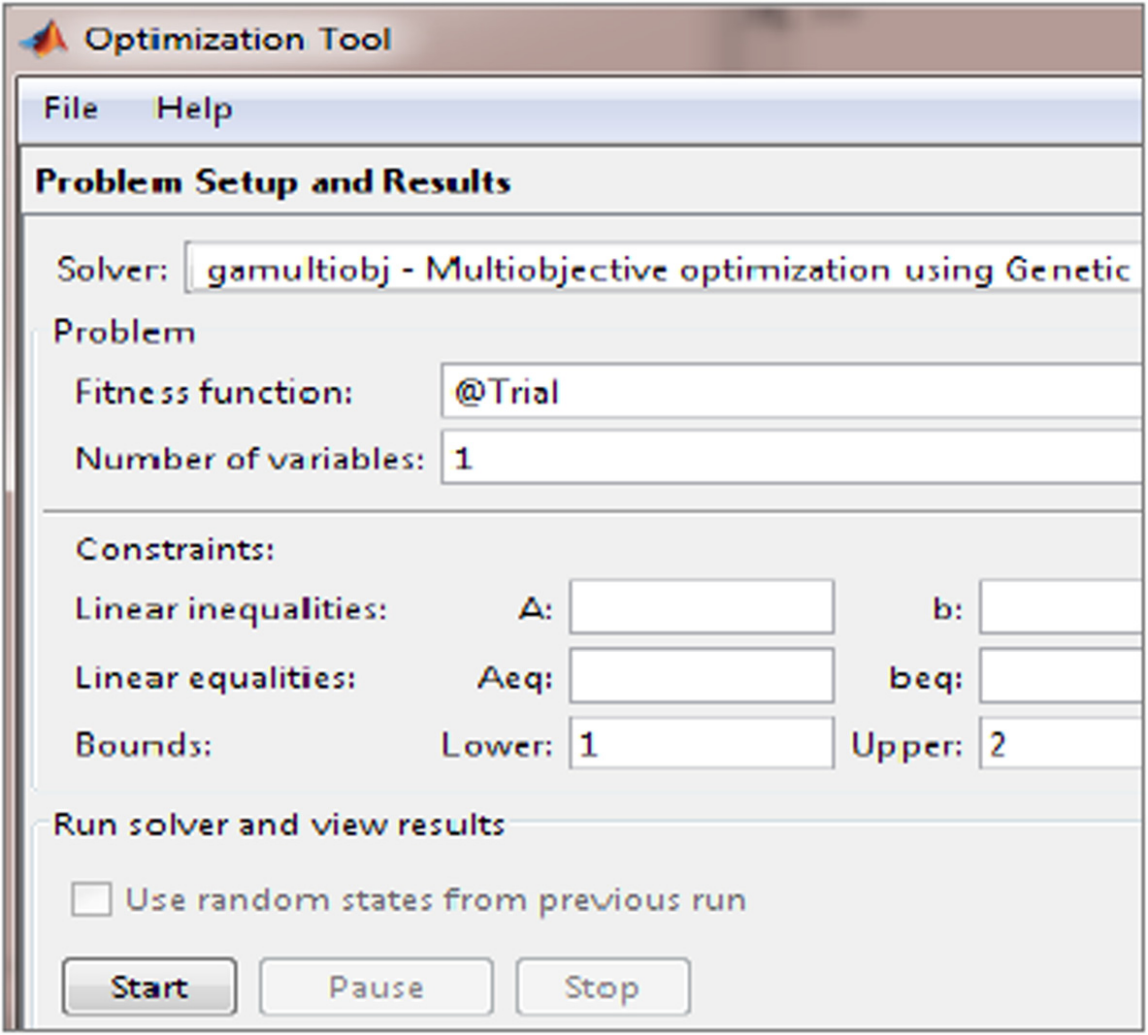
**Setting optimization parameters using MEO in Matlab.**

Optimization is maximization of imperceptibility and robustness. It refers to achieve highest balanced values of PSNR and NC. In the experimentation, Matlab tool multiobjective optimization using genetic algorithm is used as solver and ‘Trial’ function is specified as objective function as shown in Figure 4. The objective function is also called as fitness function. The scale factor K1 is passed to this function. All GA parameter setting of case 1 and case 2 is done in optimization tool.

The technique is tested for minimum, average and maximum values of K1 during experimentation with standard candidate test images Lena, peppers and lake. The experimentation is done up to maximum 10 generations. The special tests with number of generations as 5 and number of generations as 10 are carried out as shown in Table 3. The specific GA parameters used for individually for case 1 and case 2 are shown in Table 4.

**Table 3:** **Performance under varying number of generations with scale factor K1, PSNR for watermarked images (512 × 512 size) Lena, peppers and lake with NC for extracted watermark cameraman (256 × 256 size)**

**# generations**	**Test cases for scale factor(K1)**	**Watermarked image Lena**			**Watermarked image peppers**			**Watermarked image lake**		
		**K1**	**PSNR**	**NC**	**K1**	**PSNR**	**NC**	**K1**	**PSNR**	**NC**
#5	Minimum K1	1.5443	79.8611	0.9728	1.4769	93.2853	0.9791	2.5227	87.8446	0.9773
	Average K1	2.1957	65.4463	0.9890	2.2085	73.9911	0.9822	3.1875	73.0528	0.9808
	Maximum K1	4.4482	51.9741	1	4.4798	55.2266	1	4.8300	59.5709	1
#10	Minimum K1	1.5498	79.5746	0.9730	1.4769	87.2647	0.9796	2.5227	86.7532	0.9770
	Average K1	3.3328	65.8698	0.9877	2.2085	70.7811	0.9838	3.1875	73.0528	0.9808
	Maximum K1	3.3328	50.9538	1	4.4798	53.4861	1	4.8300	60.3296	1

**Table 4:** **GA parameter setting in multiobjective optimizer tool in Matlab**

**Parameter setting**	**Case: 1**	**Case: 2**
No. of generations	5	10
population size	15	15
Reproduction rate	0.8	0.8
Crossover rate	1.0	1.0
Mutation rate	0.2	0.2
Scale factor (K1)	1 to 5	1 to 5

**Figure 5:**
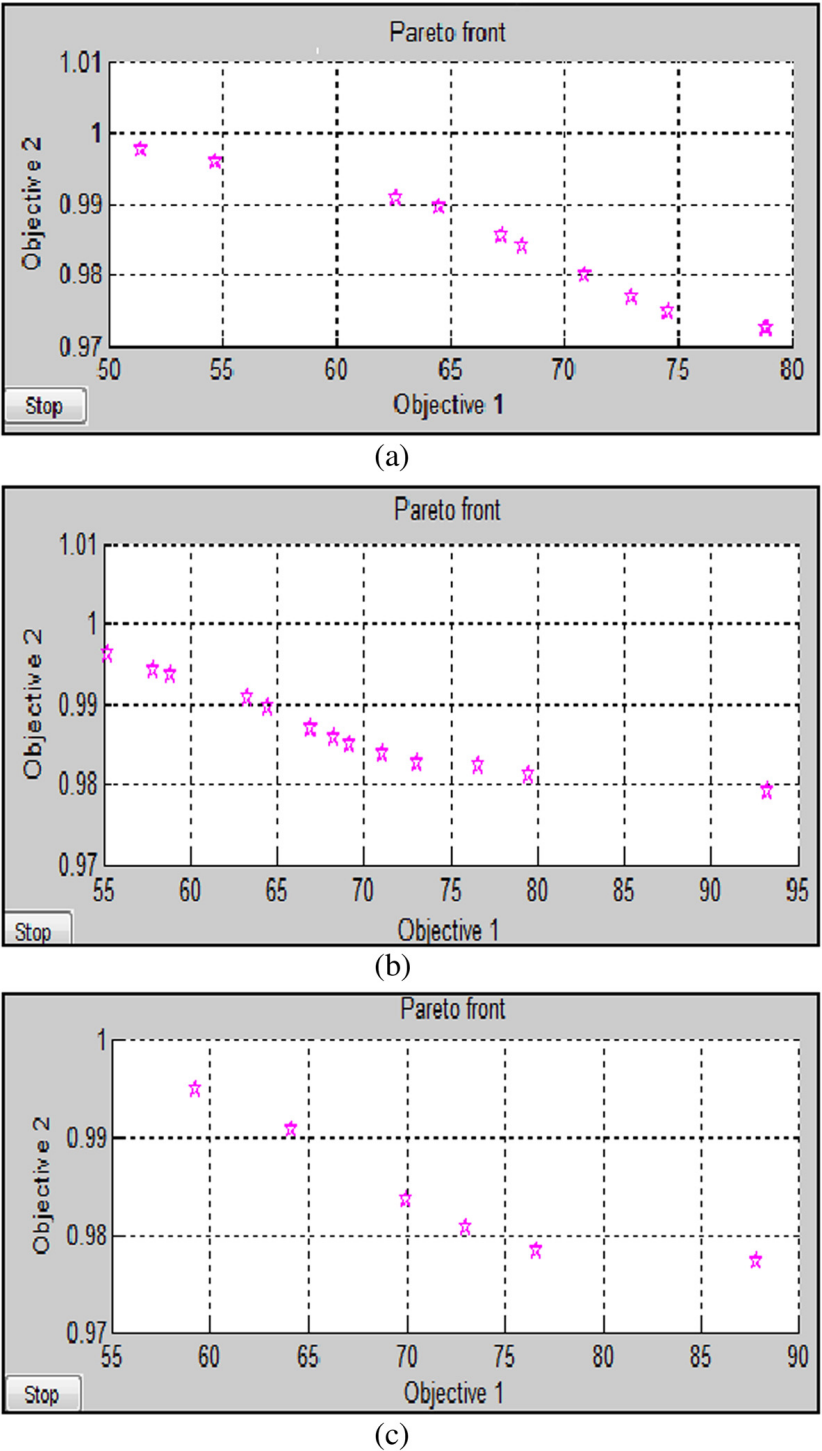
**Pareto front obtained for a) Lena b) peppers and c) lake image in GA process.**

The best values of PSNR and NC are noted for given scale factor K1 for individual cases from Pareto front graph obtained in optimization tool. The sample Pareto front graphs for Lena, peppers and lake images are shown in Figure 5. In case 1,we got PSNR from 51.9741 dBs to 79.8611 dBs and NC is from 0.9728 to 1 for Lena image. The PSNR is varied from 55.2266 dBs to 93.2853 dBs and NC is varied from 0.9791 to 1 for peppers image. Similarly, PSNR is varied from 59.5909 dBs to 87.8446 dBs and NC is varied from 0.9773 to 1 for Lake image. In case 2, we got PSNR from 50.9538 dBs to 79.5746 dBs and NC from 0.9730 to 1 for Lena image. The PSNR is varied from 53.4861 dBs to 87.2647 dBs and NC is varied from 0.9796 to 1 for pepper image. Similarly, for Lake Image, PSNR is varied from 60.3296 dBs to 86.7532 dBs, NC is varied from 0.9770 to 1. The value of scale factor is noted for every case. From experimental results it is clear that peppers image gives better performance with compared to lake and Lena images because quality factor of peppers image is better than quality factor of lake and Lena images. Here, the values of scale factor K1 are noted for minimum, average and maximum cases to have optimized values of PSNR and NC.

### Robustness test

In addition to perceptual quality the proposed technique also achived robustness under variety of attacks.

**Figure 6:**
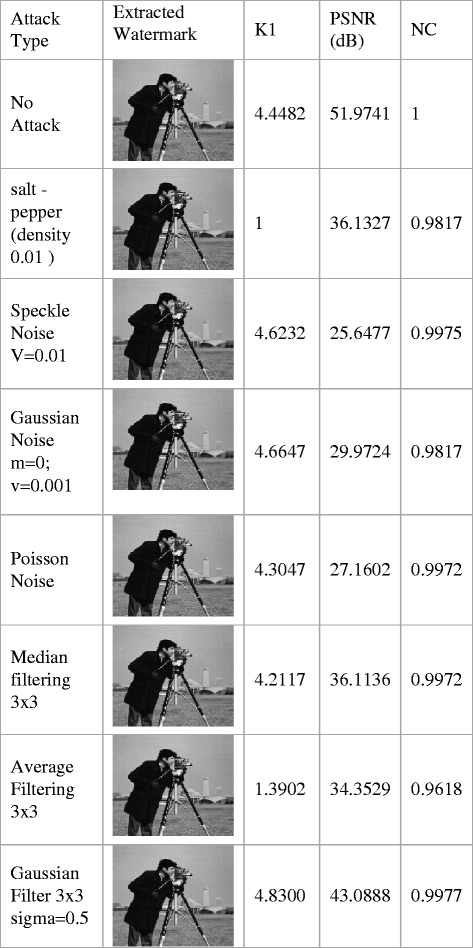
**Extracted watermark, PSNR for watermarked_image, NC for extracted_watermark with and Lena image, generations = 5 under noise addition and filtering attacks.**

**Figure 7:**
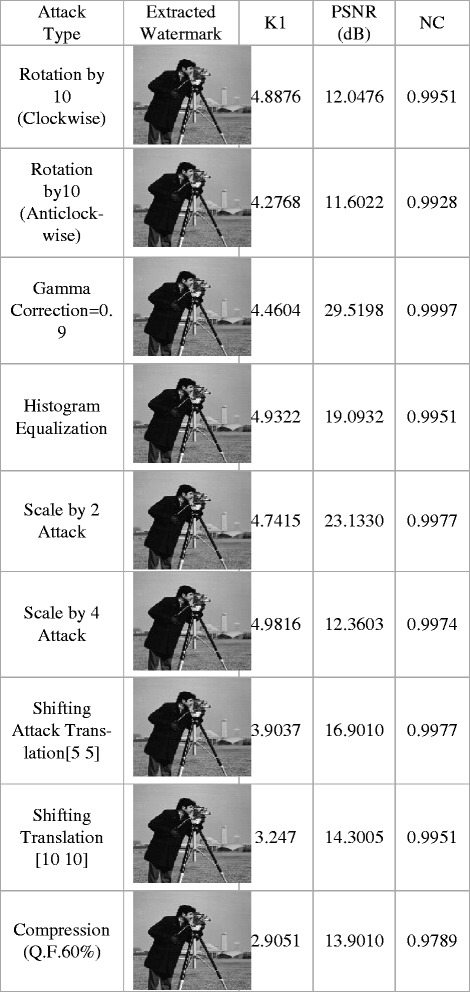
**Extracted watermark, PSNR for watermarked_image, NC for extracted_watermark with and Lena image, generations = 5 under rotation, gamma correction, histogram equalization, scale, shifting and compression attacks.**

The GA parameters for robustness test are set with number of generation as 5, population size as 15, reproduction rate as 0.8, crossover rate as 1.0, mutation rate as 0.2, bounds of scale factor K1 from 1 to 5. The score diversity plot and pare to front graph under each attack is noted from optimization tool. The robustness tests are carried for Lena image with PSNR as 51.9741 and NC as 1 at scale factor K1 as 4.4482 indicating exact recovery of watermark. The experimentation is done with watermarked image Lena under different attacks. The out coming results including extracted watermarks, PSNR, NC and K1 for watermarked image Lena, with number of generations as 5 are presented in Figures 6 and 7.

The proposed method shows significant achievement of results in all attacks under consideration namely, median filtering 3X3 , average filtering 3X3 , Gaussian filter 3 × 3 with sigma = 0.5, wiener filter 5 × 5, salt and pepper with density 0.01, speckle Noise V = 0.01, Gaussian noise m = 0, v = 0.001, Poisson noise, rotation by 5(clockwise), rotation by-5 (anti-clockwise), gamma correction = 0.9, histogram equalization, scale by 2 attack, scale by 4 attack, shifting attacks namely translation [5 5], translation [10 10] and compression attack with qualify factor (Q.F.60%).

This testing has been carried out by noting score diversity plot and pare to front observations for individual attack in multiobjective evolutionary optimizer tool of Matlab.

**Figure 8:**
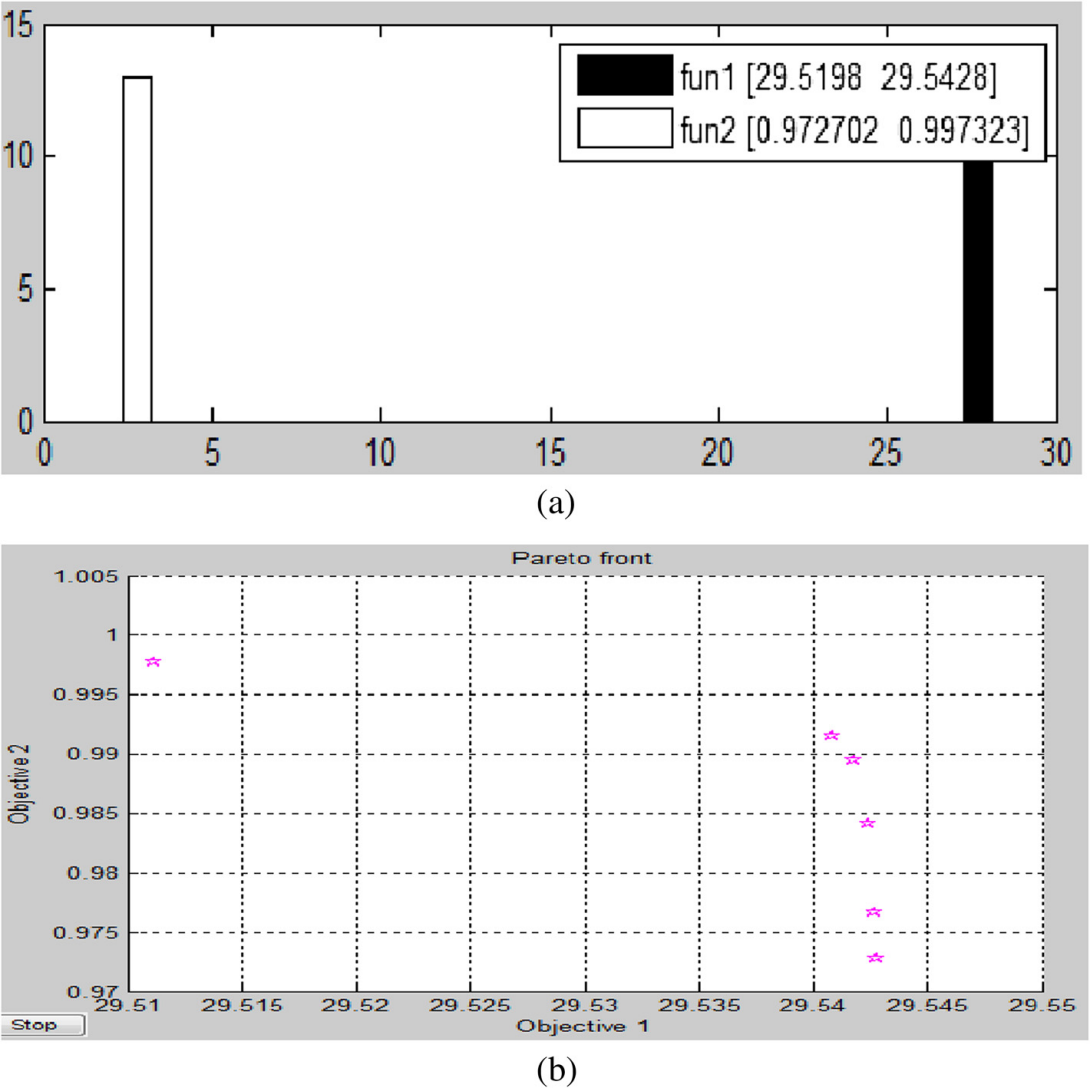
**Gamma correction attack by 0.9:a) score diversity plot b) pareto front.**

The ‘Trial’ function displays PSNR, NC and K1 for individual attack. The score diversity plot and Pareto front for gamma correction attack are shown in Figure 8. The score diversity plot shows maximum and minimum possible values. The pare to front shows paring of values of objective1 with objective 2 under individual attack.

### Comparative performance analysis

**Figure 9:**
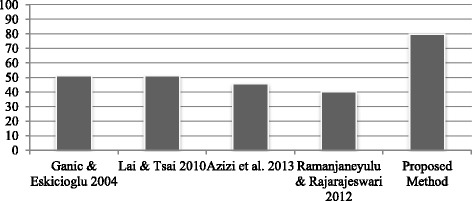
**Comparative of imperceptibility in terms of PSNR in dBs for proposed method and method (Lai & Tsai** 2010 **), method (Ganic & Eskicioglu** 2004 **), method (Azizi et al.** 2013 **) and method (Ramanjaneyulu & Rajarajeswari** 2012 **).**

The embedding capacity of proposed technique is tested with watermark of size 256 × 256 to illustrate that proposed technique supports more watermark hiding capacity than four existing hybrid methods namely method (Lai & Tsai 2010), method (Ganic & Eskicioglu 2004), method (Azizi et al. 2013) and method (Ramanjaneyulu & Rajarajeswari 2012) as shown in Table 5. The comparative performance analysis is done by considering all relevant parameters including domain used for embedding, whether method is blind or non-blind, type of cover image (grey scale or color), embedding sub-band, size of cover image and size of watermark. The proposed technique supports high capacity watermark hiding with compared to method in (Lai & Tsai 2010), method in (Ganic & Eskicioglu 2004), method in (Azizi et al. 2013) and method in (Ramanjaneyulu & Rajarajeswari 2012). The comparison of perceptual quality of method (Lai & Tsai 2010), method (Ganic & Eskicioglu 2004), method (Azizi et al. 2013) and method (Ramanjaneyulu & Rajarajeswari 2012) is shown in Figure 9. The proposed method retains the better quality of watermarked image.

**Table 5:** **Comparative of proposed method with existing method (Lai & Tsai** 2010 **), method (Ganic & Eskicioglu** 2004 **), method (Azizi et al.** 2013 **) and method (Ramanjaneyulu & Rajarajeswari** 2012 **)**

**Comparative\methods**	**Method (Lai & Tsai** 2010 **)**	**Method (Ganic & Eskicioglu** 2004 **)**	**Method (Azizi et al.** 2013 **)**	**Method (Ramanjaneyulu & Rajarajeswari** 2012 **)**	**Proposed method**
Domain Used	Hybrid DWT-SVD	Hybrid DWT-SVD	Hybrid contourlet -DCT	GA based DWT	Hybrid DWT-SVD
Category (Blind/Non blind)	Non blind	Non blind	Blind	Blind	Non blind
Type of Images (Grey/color)	Grey Scale	Grey Scale	Grey Scale	Grey Scale	Grey Scale
Embedding Sub-band/Region	HL, LH sub-bands	LL,HL,LH HH used separately	Middle frequency	LH sub-band	HL sub-band
Cover image with size	Lena 256 × 256	Lena 512 × 512	Peppers 512 × 512	Lena 512 × 512	Lena 512 × 512
Watermark with size	Cameraman 128 × 128	Cameraman 128 × 128	Binary logo 32 × 32 size	Binary logo 64 × 64	Cameraman 256 × 256

The proposed method is found more robust than method (Lai & Tsai 2010) and method (Ganic & Eskicioglu 2004) against cropping (50%), rotation (10° clockwise) Gaussian Noise (density 0.001), average filtering (3 × 3),compression (Q.F.60%), histogram equalization, Gamma correction (0.9), contrast adjustment attacks as shown in Table 6.

**Table 6:** **Comparing robustness of proposed method with method (Azizi et al.** 2013 **) and method (Ramanjaneyulu & Rajarajeswari** 2012 **)**

**Attack type**	**Method (Azizi et al. ** 2013 **)**	**Method (Ramanjaneyulu & Rajarajeswari ** 2012 **)**	**Proposed (Best case)**
Median Filter (3 × 3)	0.960	0.8130	**0.9972**
Gaussian Filter(3 × 3)	---	0.9069	**0.9977**
Average Filter (3 × 3)	---	0.6884	**0.9618**
Histogram Equalization	0.99	0.8880	**0.9951**
Gaussian Noise (Density 0.001)	0.800	0.3922	**0.9817**
Gamma Correction (0.9)	0.99	0.9983	**0.9997**
Rotation (By 10° clockwise)	---	0.5695	**0.9951**
Weiner Filtering (5 × 5)	---	0.8447	**0.9728**
Salt and Pepper Noise(0.01)	0.930	0.9263	**0.9817**
Resizing (50%)	0.960	0.6700	**0.9977**
Compression (Q.F.60%)	0.816	0.9375	**0.9789**

The proposed method is also compared with method (Azizi et al. 2013) and method in (Ramanjaneyulu & Rajarajeswari 2012) for histogram equalization, Gaussian noise (density 0.001), Gamma correction(0.9), rotation (by 10° clockwise), wiener filtering (5 × 5), salt and pepper noise (0.01), resizing (50%), compression (Q.F.60%) attacks as shown in Table 7. The significant achievement is found in normalized correlation between embedded watermark and extracted watermark for the attacks under consideration.

**Table 7:** **Comparing robustness of proposed method with method (Lai & Tsai** 2010 **) and method (Ganic & Eskicioglu** 2004 **)**

**Attack type**	**Method (Lai & Tsai** 2010 **)**	**Method (Ganic & Eskicioglu ** 2004 **)**	**Proposed (Best case)**
Cropping Attack (50%)	0.9843	0.7063	**0.9872**
Rotation Attack (10° clockwise)	0.9897	0.9091	**0.9951**
Gaussian Noise (Density 0.001)	0.9756	0.9377	**0.9817**
Average Filtering Attack (3 × 3)	0.9597	0.7047	**0.9618**
Compression Attack (Q.F.60%)	0.9772	0.9226	**0.9789**
Histogram Equalization	0.9890	0.9700	**0.9951**
Gamma Correction(0.9)	0.9994	0.9989	**0.9997**
Contrast Adjustment (Histogram method)	0.9958	0.9759	**0.9978**

In order to evaluate efficiency in terms of computation time the experimentation is carried out with proposed experimental setup. The computation time is measured by,

14 $$ \mathrm{Computation}\kern.1em \_\mathrm{time}\ \left(\mathrm{C}\mathrm{T}\right) = \mathrm{c}\mathrm{p}\mathrm{u}\kern.1em \_\mathrm{time}-\mathrm{start}\kern.1em \_\mathrm{time} $$

Where, start _time_ is the time recorded at the beginning of algorithm execution and cpu _time_ is time recorded at end of the algorithm execution.

We ran our technique with number of generations as 1, population size as 15, reproduction rate as 0.8, crossover rate as 1.0, mutation rate as 0.2, scale factor K1 as 1.5443, cover image Lena of size 512 × 512, watermark cameraman of size 256 × 256.

The method (Lai & Tsai 2010), method (Ganic & Eskicioglu 2004), method (Azizi et al. 2013) and method (Ramanjaneyulu & Rajarajeswari 2012) are run in our experimental setup using algorithmic steps proposed in those methods. The observations are listed in Table 8.

**Table 8:** **Comparative efficiency in terms of computation time(embedding plus extraction time in unit seconds)**

**Method (Lai & Tsai ** 2010 **)**	**Method (Ganic & Eskicioglu ** 2004 **)**	**Method (Azizi et al.** 2013 **)**	**Method (Ramanjaneyulu & Rajarajeswari ** 2012 **)**	**Proposed method**
3.1930	5.2900	3.5729	4.2890	3.1512

The experimental results clearly show that proposed technique is faster with compared to method (Lai & Tsai 2010), method (Ganic & Eskicioglu 2004), method (Azizi et al. 2013) and method (Ramanjaneyulu & Rajarajeswari 2012).

The observations can be summarized based on experimental demonstration:

i. The proposed technique is more robust than method (Lai & Tsai 2010), method (Ganic & Eskicioglu 2004), method (Azizi et al. 2013) and method (Ramanjaneyulu & Rajarajeswari 2012) for all attacks under considerations in HL sub-band.
ii. The proposed technique got significant achievement in perceptual quality than method (Lai & Tsai 2010), method (Ganic & Eskicioglu 2004), method (Azizi et al. 2013) and method (Ramanjaneyulu & Rajarajeswari 2012).
iii. The proposed method supports high capacity watermark embedding compared to method (Lai & Tsai 2010), method (Ganic & Eskicioglu 2004), method (Azizi et al. 2013) and method (Ramanjaneyulu & Rajarajeswari 2012).
iv. Experimentation is carried out for all minimum, average and maximum values of scale factor K1 for standard candidate images under test to know worst case, average case and best case performance of proposed method.
v. The majority of existing DWT-based image watermarking techniques are less robust to rotation and translation attacks. The proposed technique shows robustness towards rotation as well as translation attacks.
vi. The proposed technique is faster than method in (Lai & Tsai 2010), method in (Ganic & Eskicioglu 2004), method in (Azizi et al. 2013) and method in (Ramanjaneyulu & Rajarajeswari 2012).
vii. The proposed method is implemented through eight stages of security including FLT for watermark scrambling.

## Conclusions

Existing GA based techniques are relatively slow. The DWT decomposition with Haar wavelet gives better PSNR with reduced computation time compared to DWT decomposition by Symlet, db4 and db8, bior4.4 and coif5. Hence, Haar wavelet is selected for DWT decomposition to achieve better performance. We achieved improvement of quality parameters with number of generations as 5 and 10. The proposed technique achieved normalized correlation as 1 for all cover images indicating exact recovery of watermark. We got PSNR 79.8611 for Lena, 87.8446 for peppers and 93.2853 for lake images when scale factor K1 was varied from 1 to 5. The proposed technique is compared to existing methods under consideration namely, method (Lai & Tsai 2010), method (Ganic & Eskicioglu 2004), method (Azizi et al. 2013) and method (Ramanjaneyulu & Rajarajeswari 2012) and found robust against variety of attacks. The technique supports high capacity hiding and perceptually superior to method (Lai & Tsai 2010), method (Ganic & Eskicioglu 2004), method (Azizi et al. 2013) and method (Ramanjaneyulu & Rajarajeswari 2012). As Fibonacci-Lucas transformation is used, it is more secured with compared to Arnold CAT map, modified Arnold transform, Fibonacci series or generalized Fibonacci series. This technique has provided with eight layer security. In SVD, we have used multiplicative rule to improve quality parameters, whereas most of existing SVD based methods have used additive rule while embedding watermark in cover image. Majority of the existing DWT based algorithms use either or all LL, HL, LH, HH sub-bands for watermark embedding. We carefully selected HL sub-band. This technique is found more robust for rotation and translation attacks, though existing DWT based methods are less robust to these attacks. The ISO JPEG 2000 compression standard replaced DCT by DWT which is used by us. Ultimately, we are following ISO standards in our implementation. This technique is flexible and can be easily extended for color image watermarking namely RGB, YUV, YIQ, YCgCb and LUV color spaces to hide watermark in one of more color planes in HL sub-bands. The underlying technique can be extended for video watermarking. This work is in progress.
